# Comprehensive framework for interpretation of WaPOR water productivity

**DOI:** 10.1016/j.heliyon.2024.e36350

**Published:** 2024-08-15

**Authors:** Shadman Veysi, Eslam Galehban, Milad Nouri, Sina Mallah, Hamideh Nouri

**Affiliations:** a Soil and Water Research Institute, Agricultural Research, Education and Extension Organization (AREEO), Karaj, Iran; b Department of Remote Sensing and GIS, Faculty of Geography, University of Tehran, Tehran, Iran; c Water Science and Policy Coordination, DCCEEW, Australian Government, Australia

**Keywords:** Crop type, Dimensionless indices, Machine learning, Support layers, Water scarcity

## Abstract

This study presents a comprehensive framework for analyzing water productivity products provided by the FAO Water Productivity Open-access portal (WaPOR), focusing on various crops cultivated in both rainfed and irrigated areas within a semi-arid basin in Iran. Two indices, namely Gross Water Productivity (GWP) and Net Water Productivity (NWP), were introduced to quantify water productivity across crop fields. However, these indices may mislead decision-makers, because they aggregate water productivity for all crops and exacerbate the challenges posed by water scarcity. Therefore, mapping crop types seems necessary to enhance the interpretation of these indices and develop a dimensionless index for comparing different crops. The results demonstrated a fundamental change when comparing dimensionless water productivity with GWP and NWP products. Surprisingly, some pixels initially exhibiting high water productivity ranked as low water-productive pixels based on the derived dimensionless index, and vice versa. Based on dimensionless indicators, rainfed crops, particularly rainfed cereals, ranked as the most water-productive crops. The areas with dimensionless values below 0.5 warrant heightened attention to curtail non-beneficial water consumption and elevate water productivity. This research emphasizes the significance of mapping cultivation types as supplementary layers to facilitate precise, data-driven decision-making and enable comparisons of crops based on dimensionless water productivity indices.

## 1 Introduction

Water scarcity is commonly defined as a situation where demand for water surpasses available supply [1]. In a broader context, economic water scarcity arises from water mismanagement, weak governance, insufficient investment in water infrastructure, or a lack of human capacity, all of which constrain access to water [2,3]. Agricultural economic water scarcity was also defined as the lack of irrigation not due to hydrological limitations but rather because of restricted institutional and economic capacity [4].

Water scarcity is a critical limit that endangers food security in drylands and is expected to worsen due to climate change and population growth by 2050 [[5], [6], [7]]. Meeting the challenge of producing more food for more people under harsher climatic conditions will require optimizing the water-food-energy nexus, improving water productivity (WP), and expanding cropland. However, land expansion is not a practical solution in regions already experiencing water stress. Therefore, increasing WP has been recognized as a practical strategy to achieve food security [8] and aligns with the United Nations Sustainable Development Goals (SDG 6.4) [9,10].

The physical WP is simply equals the ratio of crop yield to water consumed. Multiple definitions of water productivity (for WP) are available, with another important one being economic water productivity. It can be defined as the ratio of the value of agricultural benefit to the amount of water consumed or used in the production process. This metric assesses the economic efficiency of water use in agriculture by considering the monetary return obtained from the water input [11]. Nutritional WP also offers a valuable metric for assessing WP by quantifying the nutrient output generated per unit of water used [12]. Nevertheless, physical WP, commonly referred to as “crop per drop”, remains the most widely recognized definition of water productivity.

Despite its simplicity, several challenges are associated with accurate interpretation and calculation of physical WP. One of the challenges is the misinterpretation of WP, which assumes that increasing its value leads to water savings in water-scarce regions. However, improving WP does not necessarily save water but instead can lead to an increase in water consumption, known as the Jevons’ paradox [13,14].

The scale dependency of WP is one of the main reasons causing miscalculation and misinterpretation. Since actual evapotranspiration (ET) is hardly measured at the field scale, WP is often estimated using inflow (irrigation + effective rainfall). WP is, therefore, obtained based on the water inflow in fields. In this case, the summation of water inflow volumes at fields situated in a given region may not be equal to total inflows at the catchment scale. This can be ascribed to the fact that a portion of return flows (non-consumptive recoverable deep percolation + runoff) in a field may become inflow in an adjacent field [15]. This explains why water-saving at the field scale cannot necessarily translate into water-saving at the regional scale, known as real water-saving, particularly in closed catchments. In other words, by enhancing the WP values at the field scale, one may expect WP increment but not real water-saving at the basin-scale. Therefore, this misinterpretation may result in misguided and flawed policies in water-stressed areas wherein water-saving should be at the top of the agenda.

Comparing WP of different crops cultivated in a region is another pitfall [16]. In some cases, water productivity is represented for a large area (e.g., a basin scale), which is common for policymakers to calculate regional WP by dividing the total crop production to total water use in a basin [17]. However, the numerator encompasses a broad spectrum of crops, and aggregating all crop productions to compute WP at a regional scale, may be misleading [18]. Neglecting other factors than water affecting the WP can also cause misinterpretation of WP values [19]. For instance, WP of a given crop in a specific region is affected by crop type, cultivar, climatic factors, management, soil condition, etc. [20,21]. When defining the WP gap, it is important to note that these gap values should be treated regionally, and caution should be exercised when recommending or comparing WP values obtained under different regions. WP obtained for a high-yielding humid part may not be suitable as a norm for a low-yielding hyper-arid area.

Alternative gridded datasets have been widely applied in water management to provide invaluable data in data-poor areas [22,23]. These datasets can also be helpful in determining and interpreting WP, as they provide necessary data (e.g., evapotranspiration components and biomass) and information on other factors affecting WP, such as soil, climate, topography, and management [[24], [25], [26], [27], [28], [29]]. However, uncertainties in gridded products can affect the accuracy of WP results, which can impact decision-making processes. Therefore, caution must be exercised when using gridded products to estimate WP in different basins with varying crop cultivation under rainfed and irrigated conditions.

There are limited studies on the application of gridded datasets to analyze WP. One such study, referenced as [30], suggested calculating dimensionless WP for wheat, rice, and maize on national and global scales. Initially, they merged crop dominance maps [31] with Monthly Irrigated and Rainfed Crop Areas from MIRCA-2000 [32] at a 10 km spatial resolution. Subsequently, employing satellite-derived Normalized Difference Vegetation Index (NDVI) data, they refined and disaggregated global maps of cereals to a 1 km spatial resolution. Then, they developed a formula to estimate WP based on remotely-sensed parameters such as the fraction of Absorbed Photosynthetically Active Radiation and surface albedo. Notably, their methodology relied on broad spatial and temporal scales for crop mapping, utilizing NDVI data at 10-day intervals spanning from 1998 to 2008. Consequently, their findings reflect an average condition over a decade, overlooking annual variations due to weather anomalies or anthropogenic impacts. This approach is best suited for regions with contiguous crop coverage, typified by large fields aligning with the 100 ha spatial resolution (1 km × 1 km) of crop type maps. However, it may prove inadequate for finer-scale assessments in regions with fragmented crop coverage or small fields indicative of subsistence agriculture. Furthermore, the use of satellite-derived crop maps employing unsupervised mapping techniques may introduce significant uncertainty, particularly in regions with small-scale fields and notable spatial variations in crop types.

Given these limitations, there is a pressing need for WP studies conducted at finer spatial scales to accurately capture both the spatial and annual variations in WP. This need is particularly relevant in regions like Iran, where small-scale farming predominates, and neighboring fields often exhibit diverse crop types. To address this research gap, our study aims to estimate WP within a catchment characterized by small-scale fields and annual variations, utilizing gridded products. Additionally, we introduce a dimensionless WP metric to enhance the interpretation of our results for water management purposes.

## 2 Material and methods

### 2.1 Study area

**Fig (1):**
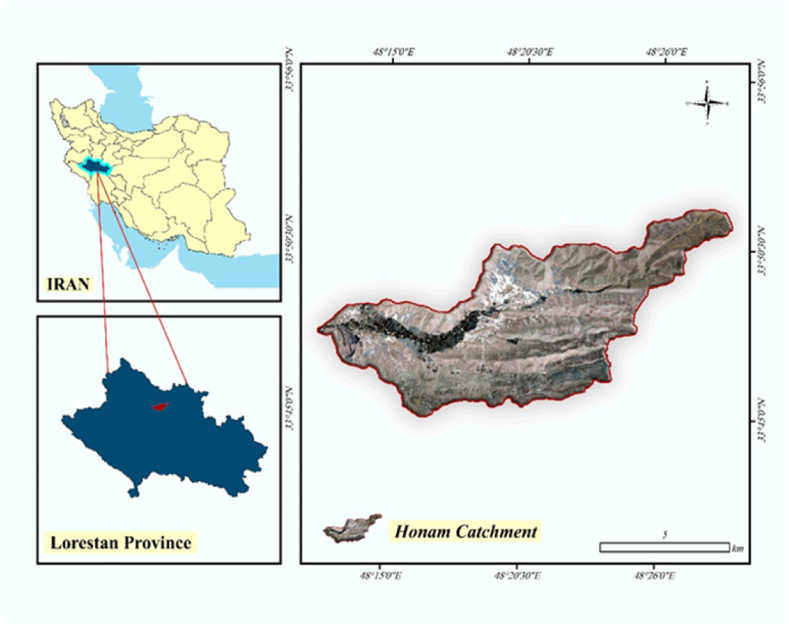
Location of the Honam catchment.

Honam is a catchment located in the Lorestan province of western Iran, covering 14,000 ha. The common crops are beans, chickpeas, sugar beet, and winter grain (i.e., wheat, barely). The average elevation of this catchment is approximately 1600 m above sea level, and the region receives an average annual precipitation of 500 mm. Fig. 1.

**Fig (2):**
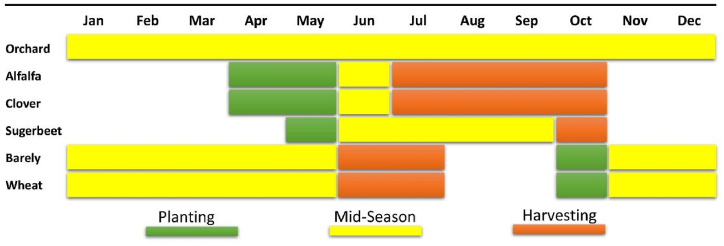
Crop calendar of the main crops in Honam catchment.

The crop calendar in the Honam region outlines the schedules for planting and harvesting the main crops. Among the various crops grown in the region wheat and barley are categorized as fall crops. Sugar beet is classified as summer crops. Alfalfa and clover are perennial crops that continue to grow for several years after planting. Orchards are considered permanent crops. It is important to note that in the Honam catchment area, there is no double-cropping on each farmland. Fig. 2 shows the crop calendar of Honam catchment for main crops.

### 2.2 Dataset and preprocessing

**Fig (3):**
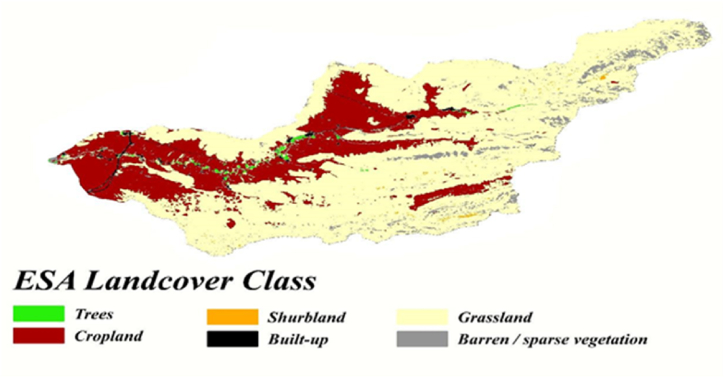
Maps of Honam 10 m resolution, Land Use Land Cover map.

Land Use and Land Cover (LULC) changes using the European Space Agency's (ESA) high-resolution satellite imagery with a spatial resolution of 10 (m) were surveyed in this research (https://www.esa-landcover-cci.org/) for detection of cropland areas. Preprocessing and extraction of cropland were performed within Google Earth Engine (GEE) Fig. 3. Therefore, crop land areas delineated from other land use categories.

Meanwhile, cropland was reprojected to a 250 (m) as target resolution. This is necessary because the Gross Water Productivity (GWP) and Net Water Productivity (NWP) were obtained from the open-access WaPOR dataset, which have a pixel size of 250 × 250 (m). The WaPOR dataset (https://wapor.apps.fao.org/) provides up-to-date information on the Near East and Africa region, including the Honam catchment. The WaPOR dataset contains several components related to water productivity, including the ratio of biomass production to total evapotranspiration, evaporation from soil, canopy transpiration, and interception. GWP and NWP take into account the water consumed solely for evapotranspiration plus interception and transpiration respectively.

(1) $$GWP=\frac{TBP}{E+T+I}$$

(2) $$NWP=\frac{TBP}{T}$$

where TBP is total biomass production in kgDM/ha. E is evaporation, T is transpiration and I is interception, all in mm. ETIa-WaPOR is derived from the ETLook model. ETLook uses a simplified Penman-Monteith model to estimate ETIa, which is based on input data such as vegetation indices, land surface temperature, and meteorological data [33]. This model separates evaporation, transpiration, and interception using equations (3), (4), (5).

(3) $$E=\frac{\Delta \left({Rn}_{soil}-G\right)+\frac{\rho_{air}C_{p}\left(e_{s}-e_{a}\right)}{{ra}_{soil}}}{\Delta +\gamma \left(1+\frac{{rs}_{soil}}{{ra}_{soil}}\right)}$$

(4) $$T=\frac{\Delta \left({Rn}_{canopy}-G\right)+\frac{\rho_{air}C_{p}\left(e_{s}-e_{a}\right)}{{ra}_{canopy}}}{\Delta +\gamma \left(1+\frac{{rs}_{canopy}}{{ra}_{canopy}}\right)}$$

(5) $$I=0.2I_{LAI}\left(1-\frac{1}{1+\frac{C_{vegPCP}}{0.2I_{LAI}}}\right)$$

where E and T (mm.day^−1^) are the evaporation and transpiration, respectively, Rn (MJ m^−2^ day^−1^) of the soil (R_n,soil_) and canopy (R_n,canopy_) is the net radiation, G (MJ m^−2^ day^−1^) is the ground heat flux, ρ*a* (kg.m^−3^) denotes the density of air, C*PP* (MJ kg^−1^°C^−1^) stands for the specific heat of air, (e*s*-e*a*) (kPa) represents the vapor pressure deficit (VPD), r*a* and r*s* (s/m) stand for the aerodynamic soil or canopy resistance, respectively, Δ (kPa°C^−1^) is the slope of the curve relating saturated water vapor pressure to air temperature, and γ denotes the psychrometric constant (kPa°C^−1^). Interception (mm.day^−1^) is a function of vegetation cover, LAI and precipitation [34]. The summary characteristics of the dataset used in the research are shown in Table .1.

**Table 1:** Data employed in this research.

Data type	symbol	Source	Spatial resolution	Temporal resolution
**Digital elevation model**	DEM	SRTM	90 (m)	*
**Land use Land Cover**	LULC	ESA	10 (m)	Annual
**Gross biomass water productivity**	GWP	WaPOR	250 (m)	Annual
**Net biomass water productivity**	NWP	WaPOR	250 (m)	Annual
**Precipitation**	*	CHIRPS	5500 (m)	Daily

### 2.3 Crop type mapping

Various remote sensing data classification methods are used to identify crop types, including Support Vector Machines [35], decision trees [36], and Random Forest [37]. Random Forest is a popular classifier that is well-suited for data distribution [38]. Therefore, the Random Forest method was used to distinct irrigated and rainfed areas and detect different crop types using vegetation indices derived Landsat-8 data coded in Google Earth Engine (GEE). Accordingly, forty-two Landsat-8 images, each with a spatial resolution of 30 m, captured in 2020 and exhibiting less than 5 % cloud coverage, were chosen from the GEE online platform to comprehensively cover the entire study area. The map from a 30-m resolution obtained from Landsat was resampled to 250 m using nearest neighbor [22,23]. It is worth noting that given the scale of field areas in our study region, upscaling the resolution from 30 m to 250 m appears reasonable, as supported by field observations. Each pixel contains either spring or winter crops, indicating that each grid is cultivated with only one crop per year. The maps for winter and spring crops were meticulously separated and integrated to derive the annual crop map. Fallow pixels were excluded to maintain accuracy. Eight vegetation indices have been used for supervising different crops, as presented in Table .2.

**Table (2):** Vegetation indices used from Landsat 8 for crop-mapping.

Index	Equation
*Normalized Difference Vegetation Index (NDVI)*	(6) $\left(R_{NIR}-{R\;}_{Red}\right)\;/\;\left(R_{NIR}+{R\;}_{Red}\right)$
*Green Normalized Difference Vegetation Index (GNDVI)*	(7) $\left(R_{NIR}-{R\;}_{Green}\right)\;/\;\left(R_{NIR}+{R\;}_{Green}\right)$
*Green Red Vegetation Index (GRVI)*	(8) $\left(R_{Green\;}-{R\;}_{Red}\right)\;/\;\left(R_{Green\;}+{R\;}_{Red}\right)$
*Green-blue vegetation index (GBVI)*	(9) $\left(R_{Green\;}-{R\;}_{Blue}\right)\;/\;\left(R_{Green\;}+{R\;}_{Blue}\right)$
*Normalized Difference Moisture Index (NDMI)*	(10) $\left(R_{NIR}-{R\;}_{Swir1}\right)\;/\;\left(R_{NIR}+{R\;}_{Swir1}\right)$
*Normalized Burn Ratio 2 (NBR2)*	(11) $\left(R_{Swir1}-{R\;}_{Swir2}\right)\;/\;\left(R_{Swir1}+{R\;}_{Swir2}\right)$
*Enhanced vegetation index (EVI)*	(12) $2.5\left(R_{NIR}-{R\;}_{Red}\right)\;\%\;(\left(R_{\text{NIR}}+6\times {R\;}_{\text{Red}}‐7.5\times {R\;}_{\text{Blue}}\right)+1$
*Green Chlorophyll Vegetation Index (GCVI)*	(13) $\left(R_{NIR}-{R\;}_{Green}\right)-1$

Bands 5, 4, 6 and 7 of Landsat-8 were represented as R_NIR_, R_Red_, R_Swir1_, and R_Swir2_, respectively, while bands 2 and 3 were represented as R_Blue_ and R_Green_, respectively.

Field sample collection is a pivotal component of the crop mapping procedure, demanding timely and meticulous execution. To ensure accuracy, we first identified hot-spot agricultural zones where various major crops are cultivated. Subsequently, 286 field sample collections were concentrated in these identified areas. Field sample collectors were equipped with appropriate instruments (e.g., GPS devices) organized for ground sampling. Samples were divided into two groups: 132 points in irrigated areas and 154 in rain-fed cultivation. 70 % of the sample data was randomly selected for training the RF classification algorithms, while the remaining 30 % was used for testing and validation. The classification performance was independently validated for each crop type using a confusion matrix. The accuracy of the classification was assessed using Producer's Accuracy (PA), User Accuracy (UA), and Kappa coefficient (K) [39]:

(14) UA=100%−CommissionError

(15) PA=100%−OmissionError

(16) $$Kappa=\frac{N\sum_{i=1}^{n}X_{ii}-N\sum_{i=1}^{n}X_{+i}X_{i+}}{N^{2}-\sum_{i=1}^{n}X_{+i}X_{i+}}$$

Where Omission Errors occur if a pixel is left out of the class type being assessed, Commission Errors happen if a pixel is incorrectly involved in the class type being assessed, *X* + i: marginal sum of the columns, *X*i+: marginal sum of the rows, N: total number of pixels in the confusion matrix, n: number of classes, and *X*ii: correctly classified pixels in each class.

### 2.4 Dimensionless water productivity

Water productivity in agriculture can be challenging due to the differences in the dimensions of the numerator and denominator [30]. To ensure a valid comparison between crops, it is essential to compare each crop within its bright and hot spots [40]. Bright and hot spots refer to crops performing better or worse than their neighbors under similar conditions. The threshold for defining better performance is arbitrary, and different studies have used different percentile values. For instance Ref. [41], used the 95th percentile by selecting the upper 5 %, while [42] defined productivity as split into four different quadrants. The process involved the detection of bright and hot pixels for each crop based on the crop type of the region, which was then used to calculate a dimensionless water productivity index (DWPI). This approach scales down features between 0 and 1. To make water productivity a useful performance indicator within this research, dimensionless GWP and NWP in Eqs. (17), (18) have been defined.

(17) $$DGWP=\frac{GWP-Hot\;pixel}{Bright\;pixel-Hot\;pixel}$$

(18) $$DNWP=\frac{NWP-Hot\;pixel}{Bright\;pixel-Hot\;pixel}$$

where GWP represents the water productivity of each pixel for each crop, 'Hot pixel' and 'Bright pixel' are the pixels with a local minimum and maximum average of 5 % of pixel water productivity for each crop, respectively.

**Fig (4):**
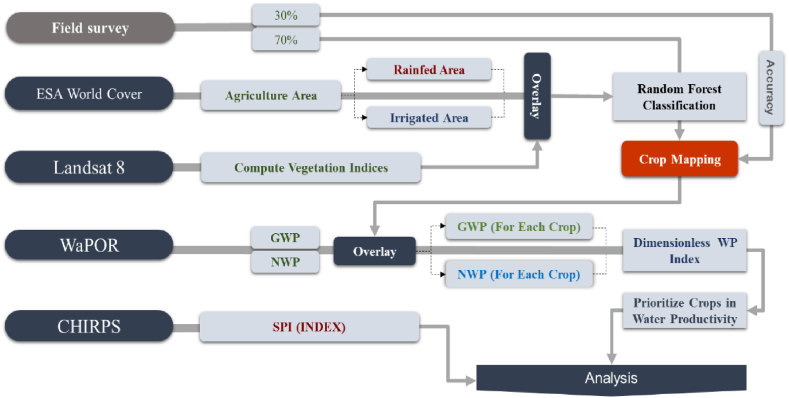
Framework of research.

DWPI is inspired by the water productivity score provided by Ref. [30], albeit with a different range. It is noteworthy that normalization is a common approach used to standardize various factors, making them dimensionless and comparable. For example, vegetation indices [43] and soil moisture content [44] are commonly formulated in a standardized manner. DWPI is a suitable for both local and regional assessments, allowing for comparisons at various scales without being influenced by specific units or environmental specifics. GWP and NWP layers were superimposed with the resize crop maps of the catchment to determine the contribution of each crop to overall productivity. Furthermore, given the potential impact of extreme values on the normalization and dimensionless representation of WP results, outliers for each crop were identified using a normal distribution. To better illustrate and clarify the methodological steps, the conceptual framework is depicted in Fig. 4.

## 3 Results and discussion

### 3.1 Mapping cultivation types and crop type

**Fig (5):**
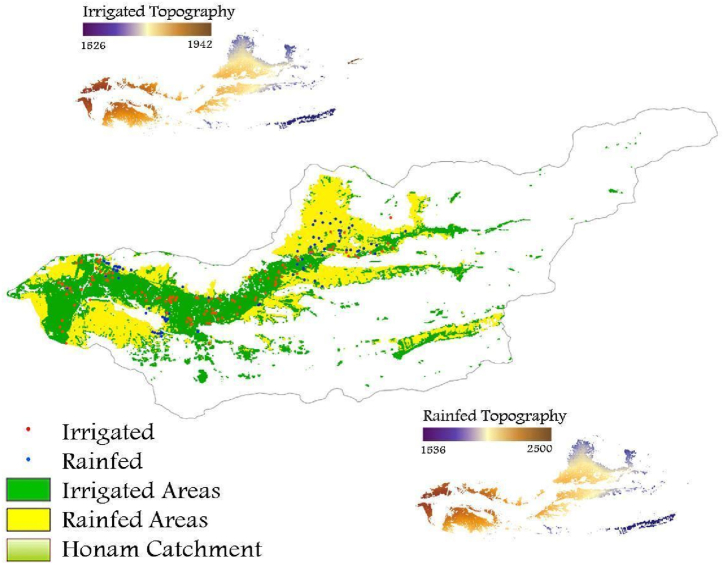
Distribution of sample points versus irrigated and rainfed areas of Honam catchment.

The map of irrigated and rainfed areas is depicted in Fig. 5, generated using the random forest method. The accuracy of detection, based on supervised point access, reached 91 % for irrigated areas and 95 % for rainfed areas. Notably, agricultural land encompasses 34.7 % of the Honam catchment, with 16.6 % and 18.2 % dedicated irrigated and rainfed cultivation, respectively. The prevalence of rainfed lands can be attributed to sufficient precipitation level in our study area.

The spatial distribution revealed a concentration of irrigated regions in the lower elevations of the catchment, while rainfed regions were predominantly situated at higher altitudes. This observed pattern may be influenced by the availability of water resources, which tend to be more abundant in lower elevations. These findings align with the work of [45], who substantiated the relationship and correlation between elevation and irrigation zones. Variations in elevation and access to water resources are pivotal factors influencing the distribution of irrigated lands [46].

**Fig (6):**
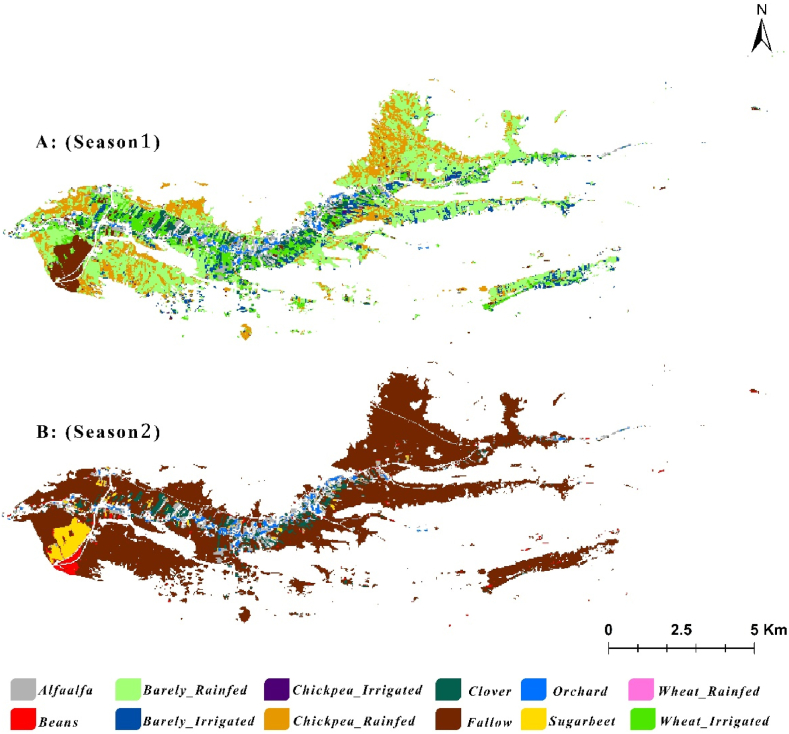
Seasonal cropping maps A) Fall/Winter cultivated crops, B) Spring/Summer cultivated crops.

**Fig (7):**
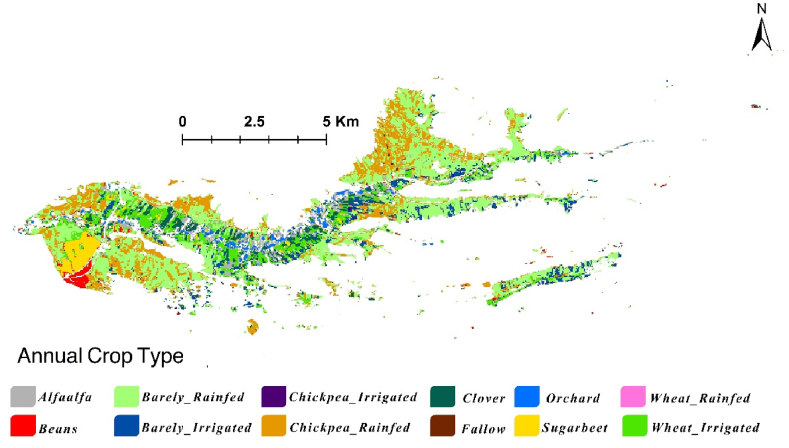
Annual cropping map.

To accurately produce the annual crop map Fig. 7, we first mapped crop patterns for two distinct seasons: season 1 (fall/winter crops) and season 2 (spring/summer crops). There are two different seasonal cropping systems in our study area, making it impractical to rely on a single annual cropping map due to significant uncertainties. Therefore, the final annual cropping map was created by overlapping the two seasonal maps depicted in Fig. 6. This approach highlights that the annual cropping pattern is a composite of different seasonal maps. It is important to note that no fields in the study area are cropped in both seasons due to phenological and resources limitations.

Fig. 7 illustrates the crop type distribution in Honam catchment. Irrigated chickpea and orchard crops were excluded from the analysis as they were relatively small. The map indicates that the area of rainfed barley is more extensive than that of other crops. However, field observations suggest that this area is a combination of both rainfed wheat and barley. Table .3 shows detail of each crop area in the catchment.

**Table (3):** Area of each crop in Honam catchment.

No	Crop	Area (ha)	No	crop	Area (ha)
1	Alfalfa	214	7	Beans	79
2	Barley Irrigated	253	8	Wheat irrigated	388
3	Clover	158	9	Chickpea Rainfed	731
4	Orchard	71	10	Wheat Rainfed	71
5	Sugar beet	122	11	Barley Rainfed	1443
6	Chickpea Irrigated	16	12	–	

Distinguishing between wheat and barley cultivations was difficult due to their similar phenology and planting schedules [47]. The study confirmed that high-resolution images are required to differentiate between cereals. Given the close alignment of planting, growing, and harvesting periods, as well as similar spectral reflection characteristics in wheat and barley, water productivity analysis for these crops was conducted by categorizing them collectively as “rainfed cereals.”

Table .4 shows the user's accuracy (UA) and producer's accuracy (PA) for each crop. According to the accuracy assessment metrics, beans had the highest UA, whereas Chickpea rainfed had the lowest significant UA. However, sugar beet had the lowest PA compared to other crops.

**Table (4):** Accuracy assessment of crop type mapping.

Crop	User Accuracy (UA)%	Producer Accuracy (PA)%	Sample Count
**Alfalfa**	0.75	0.88	28
**Barely Irrigated**	0.88	0.84	20
**Barely Rainfed**	0.78	0.86	40
**Beans**	1.00	1.00	10
**Chickpea Rainfed**	0.46	0.88	37
**Clover**	0.80	0.75	18
**Orchard**	0.73	0.73	24
**Sugar beet**	0.88	0.63	11
**Wheat irrigated**	0.79	0.81	39

Sugar beet is typically grown in various regions of Honam during the spring, but it is cultivated in small areas widely dispersed. As a result, obtaining sufficient ground samples of these classes for the network was challenging due to the difficulty of fieldwork [39]. Emphasized the importance of a sampling scheme while conducting the accuracy assessment. After calculating the Kappa statistic for the entire map, the kappa accuracy (KA) was found to be 73 %. The Kappa values were calssified as follows: <40 % as poor, 40–55 % as fair, 55–70 % as good, 70–85 % as very good, and >85 % as excellent [48]. The object-based approach yielded an average user accuracy index of approximately 79 %, suggesting that the provided cropping map is accurate. However, resampling data from a finer spatial resolution (30-m Landsat) to a coarser spatial resolution (250-m WaPOR) can introduce uncertainties. This resampling process can result in the loss of finer spatial details and obscure small-scale variations. Additionally, it can alter the spatial patterns and variability captured in the data, potentially impacting crop type mapping and water productivity results. Bilinear interpolation approach may also smooth out extremes, leading to errors. Consequnelty, we deterined the Kappa vaule for the resampled map. Accordingly, there was a Kappa value of 61 % for the resampled crop type map. Thus, despite a slight decrease in accuracy, the Kappa value obtained from the resampled cropping map, which range from 55 % to 70 %, indicate good accuracy of the results.

### 3.2 WaPOR water productivity

**Fig (8):**
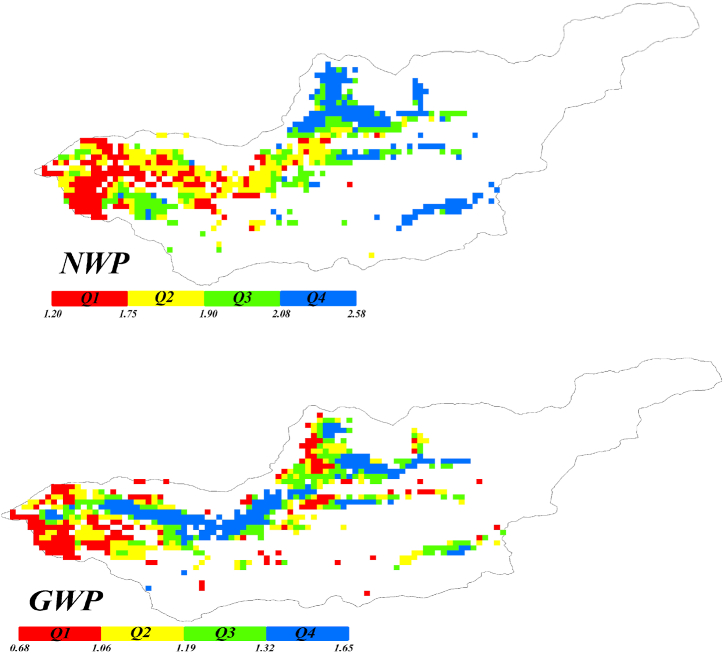
NWP and GWP of the Honam catchment.

Fig. 8 displays NWP and GWP of the WaPOR dataset. The results have been divided into quartiles, which means that their values have been split into four equal parts, with each part containing 25 % of the total values. The values ranging from 0 to 25 % are in the first class, values ranging from 25 to 50 % are in the second class, and so on for the other two types.

Overall based on raw data of WaPOR dataset, the upstream of the catchment exhibits superior water productivity compared to the downstream area based on both indices. This may be due to various factors, such as differences in field water management practices by farmers, variations in soil quality across different areas, reduced water volume downstream as it flows from upstream [49]. Emphasized that the decreased water availability may lead to reduced water productivity.

Also, Fig. 8 displays NWP being almost double GWP and a significant difference was observed. This highlights that soil evaporation (E) and interception (I) have the main role in reducing the values of water productivity. Therefore, a solution to improve water productivity in the region is to implement vapor shift [50]. To achieve this goal, management options such as increasing soil infiltration [51], enhancing water retention capacity, and leveling the lands should be considered [52].

Fig. 8 shows that some pixels with high Gross Water Productivity (GWP) values exhibit good water productivity (Q3 and Q4), whereas when using the Net Water Productivity (NWP), these same pixels are categorized as having a bad status (Q1 and Q2). When the Gross Water Productivity (GWP) is in the upper quartiles (Q3 and Q4) and the Net Water Productivity (NWP) is in the low quartiles (Q1 and Q2), the amount of total evapotranspiration (ETIa) was low. Therefore, NWP is high because maximum transpiration occurs when ETIa is low. In other words, the portion of transpiration is greater than that of evaporation (E). In addition, where the NWP proves to be suitable (Q3 and Q4) while the Gross Water Productivity GWP fails (Q1 and Q2), this means that the portion of transpiration is low as beneficial water consumption for that specific pixel.

**Fig (9):**
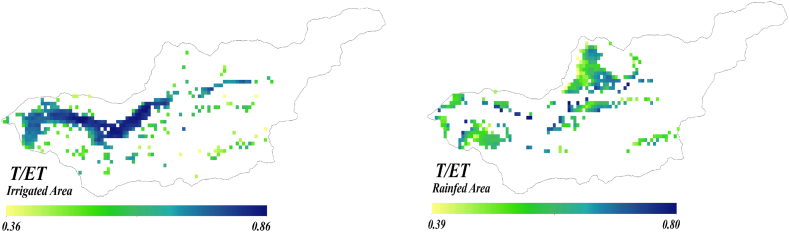
T/ET of irrigated and rain-fed areas in the Honam catchment.

Overall, these observations underscore the significance of considering the partitioning map of transpiration to evapotranspiration (T/ET) as an informative layer contingent in the particular type of cultivation for informed decision-making. Fig. 9 shows partitioning evapotranspiration (T/ET) for both irrigated and rainfed zones. Fig. 9 depicted T/ET ranges in (0.36–0.86) for irrigated areas, (0.39–0.80) for rain-fed areas. The results reported in this study align with the broad spectrum established by numerous prior global investigations [[53], [54], [55]].

**Fig (10):**
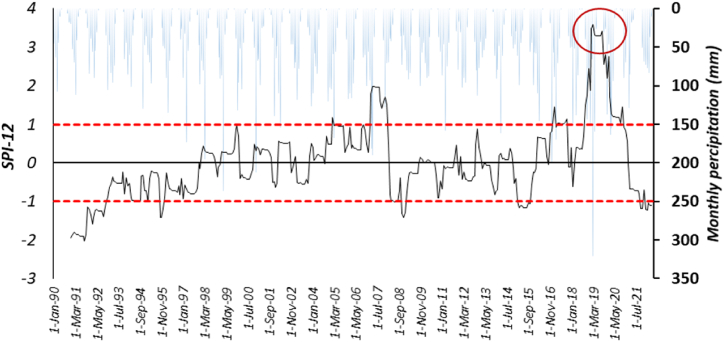
SPI variation in the Honam catchment. The upper and lower red line start with wet, and drought; second, horizontal axis shows the monthly precipitation of the region.

These results reveal no significant difference in the T/ET ratio between rain-fed and irrigated cultivation, and they oscillate within the same range. The increase in T/ET for rainfed cultivation is linked to a decline in factors such as soil moisture and rainfall events in water limited regions [56]. Under water-limited conditions, crops tend to increase their water uptake for transpiration to mitigate the impact of water restriction [57]. To further illuminate this matter, the standardized precipitation index (SPI) of the region is depicted in Fig. 10 as an indicator of droughts. This choice stems from the fact that SPI exhibits the strongest correlations between drought intensity and WP anomalies, which can be observed during or after drought events [58]. Specifically, SPI-12 was calculated based on CHIRPS precipitation dataset (1990–2021) to assess the drought status in 2020.

Fig. 10 shows that Honam experienced a very wet year in 2020, with precipitation levels reaching approximately 650 (mm). Therefore, the high rainfed productivity this year could be directly influenced by the significant amount of precipitation received. These findings are consistent with previous comparative studies conducted across different regions, which have consistently demonstrated the impacts of drought on rainfed systems in drylands [[59], [60], [61]].

### 3.3 Water productivity based on crop type

**Fig (11):**
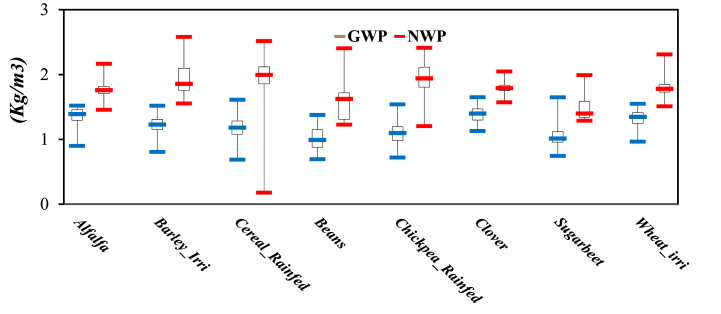
GWP and NWP for each crop.

Fig. 11 illustrates NWP and GWP for various crops. The results indicate that water productivity for rainfed crops fluctuates more than for irrigated crops. Crops with a high degree of fluctuation suggest less satisfactory management of crops and resources (such as water). Conversely, areas with low fluctuation typically experience more effective management [62]. Irrigated crops are less affected by changes because farmers have more control over water availability [63,64].

**Fig (12):**
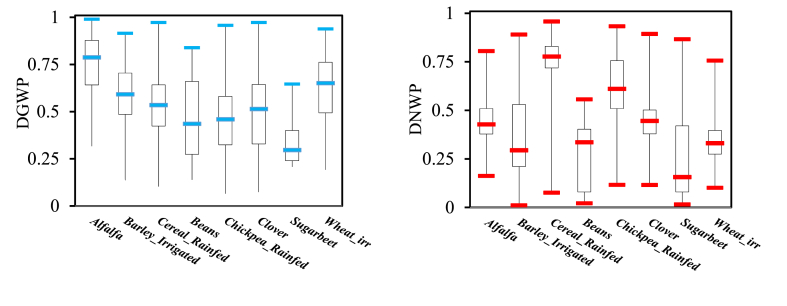
Box plot of dimensionless GWP and NWP for each crop.

Fig. 11 illustrates that the difference between the indices for alfalfa, clover, and sugar beet is minimal. When both indices yield similar values, it suggests that the proportion of evaporation (E) as non-beneficial water consumption is lower in these crops compared to others. In the categorization of water-saving strategies, this result comes from deficit irrigation practices [65] or less frequent irrigation [66], which lead to reduced soil evaporation. Also, knowing that these crops command higher prices in the market, farmers might be more inclined to implement stricter management practices, such as precise irrigation and specialized fertilizers, to protect water productivity oscillation [67,68]. Although Fig. 11 shows the water productivity of each crop, but is not easier identification which crop of efficient water use practices. Therefore, it is necessary to consider the dimensionless method for each crop Fig. 12.

Fig. 12 illustrates the dimensionless productivity indices (DGWP and DNWP) for crops that were cultivated in Honam. According to Ref. [69], the criterion for comparing the productivity of crops was set at 0.5. Therefore, any crop with an average value of less than 0.5 is considered to have poor water productivity and should be improved. Based on DWP indices, cereals and chickpeas rainfed are ranked as the most productive crops. The results show that the dimensionless indices change the ranking of crops, and consequently change water productivity interpretation and cereals rainfed shown better performance than irrigated crops.

### 3.4 Spatial mapping of DWP

**Fig (13):**
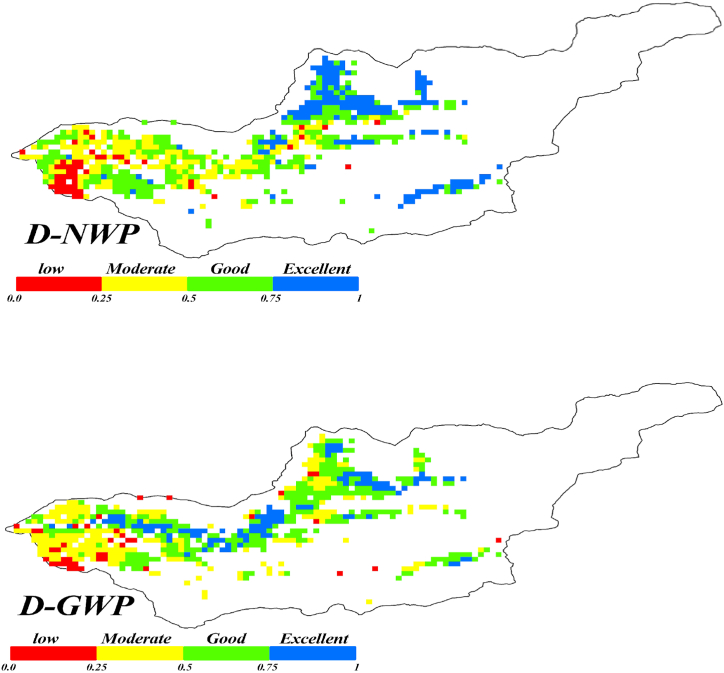
Distribution of DWPI in the Honam plain.

Fig. 13 presents a regional map of DWP, which is classified into four clusters based on the WP values. The DWP values are classified as follows: (0–0.25) for low productivity, (0.25–0.5) for moderate productivity, (0.5–0.75) for good productivity, and (0.75–1) for excellent productivity. A regional map of DWP assists in pinpointing areas where water resources are effectively utilized and highlights locations where enhancements are feasible [64].

Fig. 13, reveals significant differences compared to Fig. 9, which shows raw water productivity. Several regions depicted as exhibiting low water productivity (0–0.25) in the first quartile of Fig. 8 are reclassified as demonstrating moderate water productivity when utilizing the DWP indices.

In regions with high water productivity, there is not always a corresponding high DWP. Improving water productivity in these areas might actually lead to increased water consumption. Conversely, some areas with low water productivity may exhibit higher DWP. In regions facing water scarcity, the narrative of water productivity becomes closely intertwined with the challenge of managing water consumption. It is important to recognize that this does not always translate to reduced water consumption. Therefore, solely focusing on water productivity increases paints an incomplete picture, especially in water-scarce regions [[70], [71], [72]].

While decision-making based solely on the DNWP seems appealing due to its consideration of transpiration alone, the DGWP, which takes into account both beneficial and non-beneficial water consumption, is more practical and advisable for the decision process. The DNWP can help DGWP as it is associated with which pixel zone, and each crop better uses water, but for the final decision it is better to decide with DGWP.

Using these maps, regions characterized by low water productivity can be identified, and by correlating them with spatial data such as soil type, irrigation management, and other relevant factors, efforts can be made to uncover the underlying causes. It can be employed to distinguish between farm plots exhibiting effective and inadequate management practices.

### 3.5 Limitations

Several limitations contributed to the uncertainties in our results. Firstly, some classes were not differentiated with the desired level of accuracy due to challenges such as pronounced temporal overlap between planting, growth, and harvesting phases. This results in spectral reflection similarities between crops like wheat and barley, leading to the inevitable combination of some classes and introducing uncertainties. Moreover, another constraint was the sample quantity for each crop in crop type mapping. Acquiring sufficient samples for each crop type is challenging due to the heterogeneous distribution of crops and the costs and logistics associated with data collection. Insufficient sample sizes may introduce inaccuracies in classification algorithms, potentially impacting the accuracy and reliability of crop identification and mapping. Additionally, resampling to coordinate the WaPOR and Landsat products introduces another source of error, albeit relatively low, in the results. This process can affect the finer spatial details and alter the spatial patterns and variability captured in the data, contributing to the overall uncertainty.

Future studies can reduce uncertainties and enhance the reliability of results by exploring alternative resampling algorithms or incorporating machine learning techniques that more effectively preserve spatial details during the resampling process.

## 4 Conclusions and remarks

This study aims to demonstrate how decision-makers can apply the WaPOR water productivity (WP) dataset. The results indicate that simply presenting a raw value may be misleading. Therefore, it is essential to consider the type of cultivation (irrigated and rainfed) and crop type mapping as a supporting layers to avoid erroneous conclusions that could mislead policymakers and decision-makers. Meanwhile, when comparing two crops in the same area, it is essential to first normalize WP to make it dimensionless. In fact, the dimensionless index is one of several indicators that can be used for the physical productivity of a crop compared to another ones. Moreover, in arid and semi-arid regions, efforts to enhance water productivity may not prioritize water consumption management. This could potentially lead to an unintended increase in water use. This suggests that solely pursuing high productivity, especially in arid and semi-arid regions, can exacerbate the risk of water insecurity in these areas. Datasets such as WaPOR have the potential to offer valuable insights to help decision-makers in the water resources sector of agriculture. However, in future versions, enhancing support layers can make this dataset even more useful.

## Data availability

Data will be made available on request.

## Funding

This research received no external funding.

## CRediT authorship contribution statement

**Shadman Veysi:** Writing – original draft, Supervision, Project administration, Methodology, Formal analysis, Conceptualization. **Eslam Galehban:** Visualization, Software. **Milad Nouri:** Writing – review & editing, Resources, Investigation. **Sina Mallah:** Software, Data curation. **Hamideh Nouri:** Writing – review & editing, Methodology.

## Declaration of competing interest

The authors declare that they have no known competing financial interests or personal relationships that could have appeared to influence the work reported in this paper.
